# Self‐Supply of O_2_ and H_2_O_2_ by a Nanocatalytic Medicine to Enhance Combined Chemo/Chemodynamic Therapy

**DOI:** 10.1002/advs.201902137

**Published:** 2019-10-24

**Authors:** Shutao Gao, Yan Jin, Kun Ge, Zhenhua Li, Huifang Liu, Xinyue Dai, Yinghua Zhang, Shizhu Chen, Xingjie Liang, Jinchao Zhang

**Affiliations:** ^1^ College of Chemistry & Environmental Science Analytical Chemistry Key Laboratory of Hebei Province Chemical Biology Key Laboratory of Hebei Province Key Laboratory of Medicinal Chemistry and Molecular Diagnosis of the Ministry of Education Hebei University Baoding 071002 P. R. China; ^2^ College of Science Hebei Agricultural University Baoding 071001 P. R. China; ^3^ CAS Key Laboratory for Biological Effects of Nanomaterials and Nanosafety National Center for Nanoscience and Technology Beijing 100190 P. R. China

**Keywords:** CaO_2_, chemodynamic therapy, chemotherapy, doxorubicin, ZIF‐67

## Abstract

Combined chemo/chemodynamic therapy is a promising strategy to achieve an improved anticancer effect. However, the hypoxic microenvironment and limited amount of H_2_O_2_ in most solid tumors severely restrict the efficacy of this treatment. Herein, the construction of a nanocatalytic medicine, CaO_2_@DOX@ZIF‐67, via a bottom‐up approach is described. CaO_2_@DOX@ZIF‐67 simultaneously supplies O_2_ and H_2_O_2_ to achieve improved chemo/chemodynamic therapy. In the weakly acidic environment within tumors, CaO_2_@DOX@ZIF‐67 is broken down to rapidly release the Fenton‐like catalyst Co^2+^ and the chemotherapy drug doxorubicin (DOX). The unprotected CaO_2_ reacts with H_2_O to generate both O_2_ and H_2_O_2_. The generated O_2_ relieves the hypoxia in the tumor and further improve the efficacy of DOX. Meanwhile, the generated H_2_O_2_ reacts with Co^2+^ ions to produce highly toxic •OH through a Fenton‐like reaction, resulting in improved chemodynamic therapy.

## Graphical abstract

The hypoxic microenvironment and limited amount of H_2_O_2_ in most solid tumors severely restrict the efficacy of chemotherapy and chemodynamic therapy, respectively. Herein, a nanocatalytic medicine, CaO_2_@DOX@ZIF‐67, is synthesized via a bottom‐up approach. By virtue of simultaneously producing O_2_ and H_2_O_2_ under acidic conditions, the fabricated nanocatalytic medicine can eliminate hypoxic tumors via enhanced chemo/chemodynamic therapy.

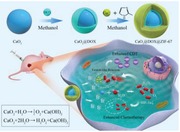

Regulation of oxidative stress in tumor tissues has been proposed as an effective strategy for cancer treatment using methods such as photodynamic therapy (PDT),^1^ sonodynamic therapy (SDT),^2^ and chemodynamic therapy (CDT).^3^ These strategies can be classified into nanocatalytic therapeutic modalities, which are triggered by light, metal catalyst and ultrasonic, respectively. Thereinto, only CDT is dependent on chemical‐stimuli‐activated.^4^ Recently, CDT has been extensively explored due to its advantages of high therapeutic specificity and low invasiveness.^5^ During the CDT process, endogenous H_2_O_2_ disintegrates into hydroxyl radicals (•OH) in the presence of metal catalysts (e.g., Fe, Mn, Cu, Co, etc.) via an intratumoral Fenton or Fenton‐like reaction.^6^ As the most harmful reactive oxygen species (ROS), •OH has a high electrochemical oxidative potential (*E*(•OH/H_2_O) = +2.80 V), which can induce cancer cell death through oxidative damage of lipids, proteins and DNA.^7^ Hence, CDT has been regarded as a promising strategy for cancer treatment. However, the limited concentration of H_2_O_2_ in tumor cells (50 × 10^−6^ to 100 × 10^−6^ m) has restricted the therapeutic efficacy of CDT.^5^, ^8^ Thus, many efforts have been devoted to increasing the intratumoral H_2_O_2_ level with various H_2_O_2_‐generating agents, such as glucose oxidase,^9^ β‐lapachone,^10^ and Au nanoparticles.^11^

In addition, to improve the antitumor effect, more and more therapeutic modalities, such as chemotherapy,^12^ starvation therapy,^13^ gas therapy,^14^ photothermal therapy,^15^ and PDT,^16^ have been used in combination with CDT to address different targets. The anthracycline class antibiotic doxorubicin (DOX) has been widely used for the treatment of a range of cancers.^17^ In aerobic conditions, DOX can elicit the production of superoxide radicals (O_2_^•−^) by activating the nicotinamide adenine dinucleotide phosphate (NADPH) oxidases and further catalyzing the reaction of NADPH and oxygen (O_2_).^18^ O_2_^•−^ can induce cancer cell death via oxidative damage to cellular components, which also increases the sensitivity of tumor cells to DOX.^19^ Alternatively, superoxide dismutase (SOD) can convert the O_2_^•−^ to H_2_O_2_,^20^ which is the substrate of CDT. However, the hypoxic microenvironment of most solid tumors limits the therapeutic efficacy of DOX. Hence, alleviating the hypoxia in the tumor microenvironment may augment the sensitivity of tumor cells to DOX and also improve the therapeutic efficacy of CDT.

Owing to its high biocompatibility and efficient O_2_‐evolving ability, calcium peroxide (CaO_2_) has previously been utilized as an O_2_ production material (Equation (1)) for improving PDT efficiency^21^ and overcoming the hypoxia‐induced DOX resistance in solid tumors.^22^ Moreover, CaO_2_ can dissolve in water to form H_2_O_2_ under acidic conditions (Equation (2)).^23^ Since CaO_2_ can simultaneously produce O_2_ and H_2_O_2_, it has great potential compared to other H_2_O_2_‐generating materials to achieve enhanced chemo/chemodynamic therapy of hypoxic tumors. Nevertheless, the aqueous instability of CaO_2_ might be a major obstacle to employing it as provider of O_2_ and H_2_O_2_. Therefore, it is highly desirable to search for a nanocarrier that can avoid premature decomposition of CaO_2_

(1) $${\mathrm{CaO}}_{2}\;\;+\;\;H_{2}O\to \frac{1}{2}O_{2}\;\;+\;\;\mathrm{Ca}{\left(\mathrm{OH}\right)}_{2}$$

(2) $${\mathrm{CaO}}_{2}\;\;+\;\;2H_{2}O\to H_{2}O_{2}\;\;+\;\;\mathrm{Ca}{\left(\mathrm{OH}\right)}_{2}$$

Herein, we designed a nanocatalytic medicine for coencapsulation of DOX and CaO_2_ within a cobalt‐based metal–organic framework (ZIF‐67). The zeolitic imidazolate framework ZIF‐67 is stable in physiologically neutral conditions, and can therefore prevent CaO_2_ from reacting prematurely. However, ZIF‐67 should decompose in the acidic environment within tumor and rapidly release Co^2+^, which can then catalyze the decomposition of H_2_O_2_ to produce •OH through a Fenton‐like reaction^24^ to achieve efficient CDT. As illustrated in **Scheme** 1, CaO_2_ nanoparticles were first prepared with PEG‐200 as solvent and template. Then, DOX was coated onto the surface of the CaO_2_ via coordination between DOX and Ca to form CaO_2_@DOX. To further isolate and protect the CaO_2_@DOX from external water, ZIF‐67 was finally constructed on the surface of CaO_2_@DOX by an in situ synthesis method. This yielded the pH‐responsive nanocatalytic medicine CaO_2_@DOX@ZIF‐67. The outermost ZIF‐67 layer will be disassembled in the acidic environment of tumor due to its pH sensitivity, resulting in rapid release of Co^2+^ and DOX. At the same time, the unprotected CaO_2_ will react with water to produce O_2_ and H_2_O_2_. The generated H_2_O_2_ will then be further catalyzed by Co^2+^ ions to produce highly toxic •OH through a Fenton‐like reaction, while the generated O_2_ will improve the efficacy of DOX by alleviating the hypoxic conditions in the tumor. Therefore, this nanocatalytic medicine provides a novel strategy for improving the effectiveness of combined chemo/chemodynamic therapy.

**Scheme 1:**
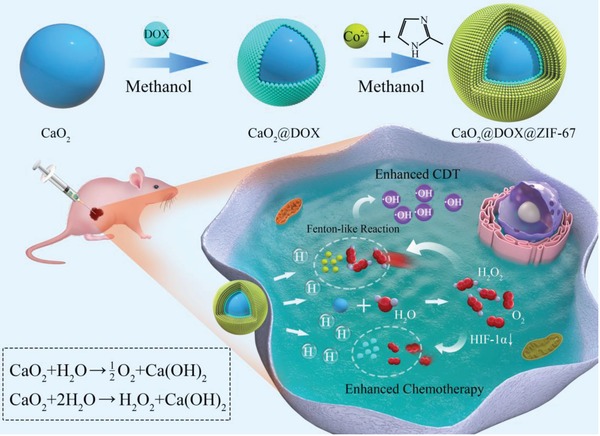
Schematic of the CaO_2_@DOX@ZIF‐67 synthetic process and the enhancement of combined chemo/chemodynamic therapy by CaO_2_@DOX@ZIF‐67 in a tumor cell.

The nanocatalytic medicine CaO_2_@DOX@ZIF‐67 was synthesized by a bottom‐up approach. First, CaO_2_ was prepared via a hydrolysis–precipitation procedure reported in the literature.^25^ Second, DOX was immobilized on the surface of CaO_2_ to form CaO_2_@DOX by coordination reaction.^26^ After modification with DOX, the nanoparticles had a more uniform morphology (Figure S1a,b, Supporting Information) and their diameters were larger (Figure S1c, Supporting Information). In addition, compared with free DOX, the UV–vis absorption spectrum of CaO_2_@DOX exhibited a significant red‐shift and two bands were observed at around 550 and 590 nm, which are characteristic of the Ca‐DOX complex (Figure S1d, Supporting Information).^26^ Finally, ZIF‐67 was constructed on the surface of CaO_2_@DOX by an in situ synthesis method. Scanning electron microscopy (SEM) and transmission electron microscopy (TEM) images of CaO_2_@DOX@ZIF‐67 revealed monodisperse nanospheres with a mean diameter of 200 nm (**Figure** 1a,b). This was also confirmed by dynamic light scattering (Figure S1c, Supporting Information). Figure 1c shows the elemental mapping of CaO_2_@DOX@ZIF‐67, which indicates that Ca, O, Co, N, and C are homogeneously distributed in CaO_2_@DOX@ZIF‐67. Powder X‐ray diffraction (PXRD) analysis of CaO_2_ and CaO_2_@DOX@ZIF‐67 is presented in Figure 1d. The as‐prepared CaO_2_ exhibited distinct peaks (2θ) at 30.1°, 35.6°, and 47.3°, which were identical with the values reported in the literature for CaO_2_ (card number 03‐0865).^27^ After encapsulation by DOX and ZIF‐67, the typical diffraction peaks indexed to CaO_2_ are not obvious, which might be ascribed to the low crystallinity of CaO_2_. In contrast, ZIF‐67 is highly crystalline, and we can observe its dominant peaks in CaO_2_@DOX@ZIF‐67.^28^ As shown in Figure 1e, the BET surface area of CaO_2_@DOX@ZIF‐67 was calculated to be 508.8 m^2^ g^−1^, which is much lower than that of ZIF‐67 (1492.5 m^2^ g^−1^). X‐ray photoelectron spectroscopy (XPS) was further used to characterize the chemical composition and chemical state of CaO_2_@DOX@ZIF‐67. The XPS survey spectrum showed that CaO_2_@DOX@ZIF‐67 is composed of Ca, O, Co, N, and C (Figure 1f). As shown in Figure 1g, the high‐resolution spectrum of Ca 2p displayed two characteristic peaks at 346.4 eV (Ca 2p3/2) and 350.1 eV (Ca 2p1/2), indicating the presence of Ca^2+^.^29^ The photoelectron peak at 533.1 eV of O1s can be attributed to O_2_^2−^,^30^ which is the other component of CaO_2_ (Figure 1h). Figure 1i displays two main photoelectron peaks at 780.8 and 796.4 eV, which can be assigned to Co 2p3/2 and Co 2p1/2, respectively. The energy gap between the main peak of Co 2p and the satellite peak (sat. 1, 785.8 eV) is 5.0 eV, which suggests that Co(II) is the main form in CaO_2_@DOX@ZIF‐67.^31^ All the above results confirmed that the CaO_2_@DOX@ZIF‐67 nanoparticles have been successfully constructed.

**Figure 1:**
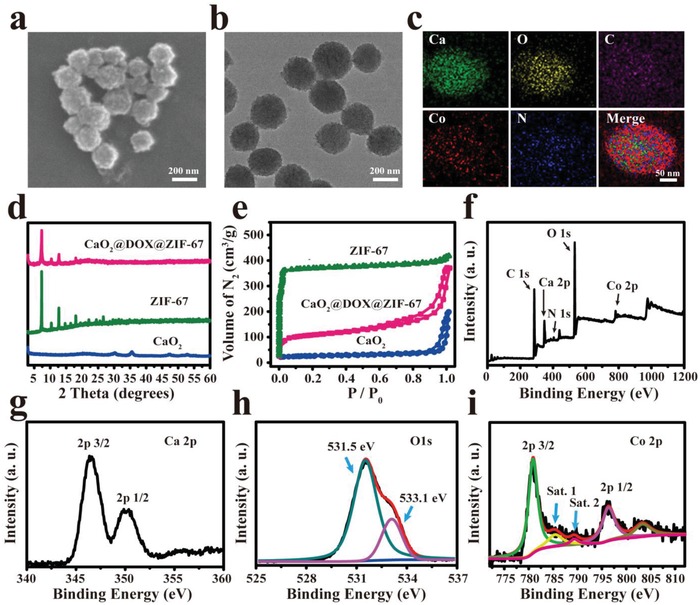
Characterization of CaO_2_@DOX@ZIF‐67. a) SEM and b) TEM images of CaO_2_@DOX@ZIF‐67; c) Elemental mapping of Ca, O, C, Co, and N in CaO_2_@DOX@ZIF‐67; d) XRD pattern of CaO_2_, ZIF‐67, and CaO_2_@DOX@ZIF‐67; e) nitrogen adsorption–desorption isotherms of CaO_2_, ZIF‐67, and CaO_2_@DOX@ZIF‐67; f) XPS spectrum ofCaO_2_@DOX@ZIF‐67; g) XPS high‐resolution spectrum of Ca 2p; h) XPS high‐resolution spectrum of O 1s; i) XPS high‐resolution spectrum of Co 2p.

UV–vis spectrophotometry was used to determine the loading of DOX in CaO_2_@DOX@ZIF‐67 and its release behavior. According to the standard curve (Figure S2, Supporting Information), the loading capacity of DOX was quantitatively determined to be 5.2 wt%. Even in CaO_2_@DOX, the loading efficiency of DOX was only 7.2%. This low loading capacity might be because the coordination sites on surface of the CaO_2_ were completely occupied. The release of DOX from CaO_2_@DOX@ZIF‐67 was measured in buffers of different pH. As shown in **Figure** 2a, there was no release of DOX at pH 7.4, and even at pH 6.5, the cumulative release of DOX was only 30%. In contrast, once the pH value was lowered to 5.0, more than 90% of the DOX was released within 15 min. These results indicated that the release of DOX is triggered by pH, which could be ascribed to the pH‐sensitivity of ZIF‐67. Figure S3 (Supporting Information) showed that ZIF‐67 could quickly decompose and release Co^2+^ within 2 min at pH 5.0. Then, the stability of CaO_2_@DOX@ZIF‐67 in serum and phosphate buffered saline (pH 7.4) was also investigated using DLS. Figure S4 (Supporting Information) showed that no detectable changes in particle size was observed within 24 h.

**Figure 2:**
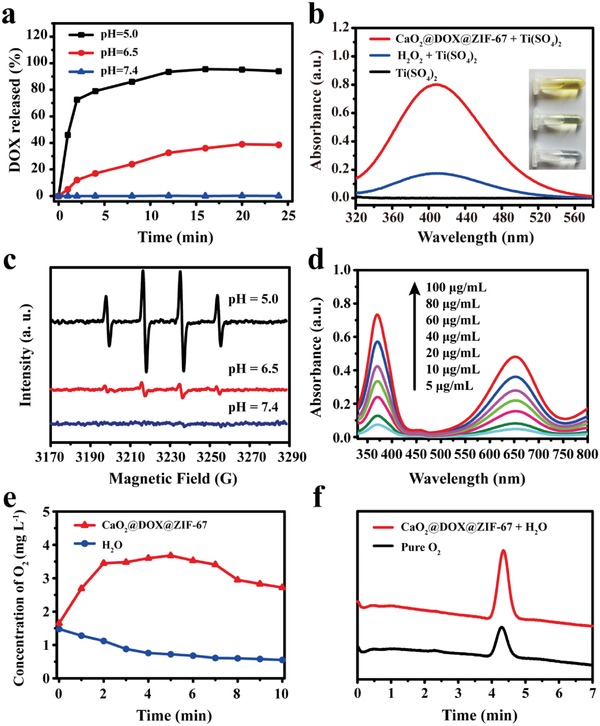
a) The release of DOX from CaO_2_@DOX@ZIF‐67 at pH 5.0, 6.5, and 7.4; b) absorbance spectrum of the titanium peroxide complex in the presence of CaO_2_@DOX@ZIF‐67 or H_2_O_2_ (determined by H_2_O_2_ assay kit); c) ESR spectra of CaO_2_@DOX@ZIF‐67 at different pH values with 5,5‐dimethyl‐1‐pyrroline N‐oxide (DMPO) as the spin trap; d) UV–vis absorbance spectra of ox‐TMB in the presence of various concentration of CaO_2_@DOX@ZIF‐67 at pH 5.0, 37 °C after 10 min; e) oxygen concentration in 20 mL deoxygenated acetate buffer (pH = 5.0) after adding 20 mg of CaO_2_@DOX@ZIF‐67 (deoxygenated water was used as the control); f) GC spectrum using thermal conductivity detector to analyze the evolved gas from CaO_2_@DOX@ZIF‐67.

After the decomposition of ZIF‐67, the unprotected CaO_2_ is predicted to react with water to produce O_2_ and H_2_O_2_. The generation of H_2_O_2_ arising from the reaction of CaO_2_ and water was evaluated using an H_2_O_2_ assay kit. As shown in Figure 2b, more yellow titanium peroxide complex (TiO_2_^2+^) was obtained in the presence of CaO_2_@DOX@ZIF‐67. The next prediction is that the generated H_2_O_2_ will further react with Co^2+^ ions released from ZIF‐67 through a Fenton‐like reaction to produce highly toxic •OH. To prove this, we used 5,5‐dimethyl‐1‐pyrroline N‐oxide (DMPO), a •OH trapping agent. The production of •OH was measured by electron spin‐resonance (ESR) spectrometry, in which the DMPO/•OH adduct presents a characteristic 1:2:2:1 four‐line signal.^7^ Interestingly, the signal intensity of the adduct is dependent on the pH. As shown in Figure 2c, the ESR signal was intensified when the pH value was lowered from 6.5 to 5.0. However, no ESR signals are observed at pH 7.4. These results further confirmed that CaO_2_@DOX@ZIF‐67 is stable in physiologically neutral conditions, while under acidic conditions, •OH radicals are effectively generated by Co^2+^‐catalyzed decomposition of the produced H_2_O_2_ through a Fenton‐like reaction. As control, the production of •OH was also measured in the presence of CaO_2_@DOX and ZIF‐67 + H_2_O_2_, respectively. The results further demonstrated that Co^2+^ ions acted as the necessary Fenton‐like reaction catalyst driving generation •OH radicals (Figure S5, Supporting Information). In addition, the generation of •OH was further evaluated at pH 5.0 using 3,3′,5,5′‐tetramethylbenzidine (TMB), which is oxidized by •OH to form ox‐TMB.^32^ As shown in Figure 2d, the UV–vis absorption spectra revealed that the production of ox‐TMB was dependent on the concentration of CaO_2_@DOX@ZIF‐67. In order to quantitatively determine the amount of H_2_O_2_ generated from CaO_2_@DOX@ZIF‐67, the absorbance values of the ox‐TMB were measured at 370 nm and a standard curve was plotted (Figure S6, Supporting Information). According to the standard curve, 58.4 µmol of H_2_O_2_ is produced by 1 mg of CaO_2_@DOX@ZIF‐67. This would greatly improve the efficacy of CDT.

The amount of oxygen generated by CaO_2_@DOX@ZIF‐67 was also determined. Twenty milligrams of CaO_2_@DOX@ZIF‐67 were added to 20 mL of buffer solution (pH 5.0) and the concentration of dissolved oxygen was monitored by a portable oxygen meter in real time. As shown in Figure 2e, compared with degassed water, the solution of CaO_2_@DOX@ZIF‐67 had a higher oxygen concentration (78 µmol g^−1^), which would improve the efficacy of DOX treatment. In order to visualize the formation of oxygen bubbles, a video was taken of the reaction of CaO_2_@DOX@ZIF‐67 with H_2_O at pH 5.0 (Video S1 is available in the Supporting Information). In addition, the exclusive formation of O_2_ was confirmed using gas chromatography (GC). Figure 2f shows that the gas generated from CaO_2_@DOX@ZIF‐67 had the same retention time as pure O_2_. These results indicate the excellent and specific O_2_‐generating capacity of CaO_2_@DOX@ZIF‐67. In summary, our data demonstrate that CaO_2_@DOX@ZIF‐67 has many properties which hold promise for enhanced chemo/chemodynamic therapy.

Encouraged by the efficient production of •OH and O_2_, we further investigated the in vitro anticancer effect of CaO_2_@DOX@ZIF‐67. The viability of MCF‐7 cells treated with CaO_2_@DOX@ZIF‐67 was evaluated by standard methyl thiazolyl tetrazolium (MTT) assay. As shown in **Figure** 3a, the viabilities of MCF‐7 cells are highly dependent on the dosage of CaO_2_@DOX@ZIF‐67 and the pH value. At the same concentration of CaO_2_@DOX@ZIF‐67, the cytotoxic effect was greater under slightly acidic conditions (pH 6.5) than under neutral conditions (pH 7.4). These results indicate that the cytotoxic effect of CaO_2_@DOX@ZIF‐67 is mainly triggered by acidic conditions. The colocalization assay demonstrated the acidic condition was mainly provided by the lysosome of MCF‐7 cells (Figure S7, Supporting Information).

**Figure 3:**
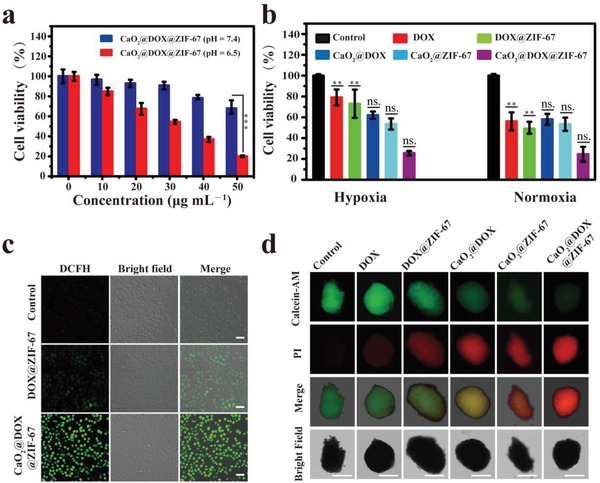
a) The cytotoxicity induced by different concentrations of CaO_2_@DOX@ZIF‐67 at pH 7.4 and 6.5 (***p < 0.001); b) the cytotoxicity induced by various treatments under normoxic/hypoxic conditions at pH 6.5 (**p < 0.01 for DOX and DOX@ZIF‐67‐group at hypoxia condition versus normoxia condition; no significant difference for CaO_2_@DOX, CaO_2_@ZIF‐67, and CaO_2_@DOX@ZIF‐67 between hypoxia and normoxia condition, *t* test); c) confocal microscopy images of MCF‐7 cells treated with 50 µg mL^−1^ CaO_2_@DOX@ZIF‐67 for 12 h and then incubated with DCFH‐DA for 30 min (scale bars represent 50 µm); d) representative images of live/dead cell assays in MCF‐7 MCTSs with various formulations treatments. Green, live cell. Red, dead cell. Scale bars are 200 µm.

As expected, the cytotoxic effect of CaO_2_@DOX@ZIF‐67 on MCF‐7 cells is independent of the concentration of oxygen owing to the capacity of CaO_2_@DOX@ZIF‐67 to relieve hypoxia. Figure 3b shows that CaO_2_@DOX@ZIF‐67 exhibited similarly high levels of cytotoxicity toward MCF‐7 cells under both normoxic (21% O_2_) and hypoxic (1% O_2_) conditions. The cytotoxic effects of CaO_2_@DOX and CaO_2_@ZIF‐67 on MCF‐7 cells were also independent of the oxygen concentration. In contrast, the viability of cells treated with free DOX or DOX@ZIF‐67 is clearly higher under hypoxic conditions than under normoxic conditions. All the above results confirm that the oxygen generated by CaO_2_ is beneficial for improving the efficacy of DOX. The excellent cytotoxicity of CaO_2_@DOX@ZIF‐67 compared to the other groups can be ascribed to the combined chemo/chemodynamic treatment effect.

To verify the effective intracellular production of •OH, we used the ROS fluorescence probe 2′,7′‐dichlorodihydrofluorescein diacetate (DCFH‐DA) (Figure 3c). Compared with the control group, DOX@ZIF‐67‐treated MCF‐7 cells showed a low level of fluorescence, which can be ascribed to conversion of endogenous intracellular H_2_O_2_ by Co^2+^ to generate •OH. However, strong green fluorescence was observed in MCF‐7 cells treated with CaO_2_@DOX@ZIF‐67. These results further indicate that CaO_2_@DOX@ZIF‐67 can significantly increase the intracellular level of H_2_O_2_ owing to the presence of CaO_2_, and the H_2_O_2_ then disintegrates to yield •OH through a Fenton‐like reaction catalyzed by Co^2+^ ions.

In addition, the combined treatment effect of CaO_2_@DOX@ZIF‐67 was further evaluated in MCF‐7 derived multicellular tumor spheroids (MCTSs) using alive‐dead cell staining assay with calcein acetoxymethyl ester (calcein AM, green fluorescence) and propidium iodide (PI, red fluorescence) (Figure 3d). Compared with the control MCTSs (treated with PBS), the MCTSs treated with CaO_2_@DOX@ZIF‐67 showed a drastic increase in red fluorescence resulting from severe cell apoptosis. Statistical analysis data showed that the intensity of red fluorescence increased about 31‐fold, and green fluorescence reduced to 1/10 compared to the control group (Figure S8, Supporting Information). In comparison, only partial apoptosis was observed in the MCTSs incubated with free‐DOX, DOX@ZIF‐67, CaO_2_@DOX, and CaO_2_@ZIF‐67. These results are in accordance with the MTT assay, and further demonstrate the combined chemo/chemodynamic treatment effect of CaO_2_@DOX@ZIF‐67.

The promising antitumor effect in vitro encouraged us to further evaluate the combined chemo/chemodynamic therapeutic performance of CaO_2_@DOX@ZIF‐67 against MCF‐7 breast tumor xenografts in nude mice. MCF‐7 tumor‐bearing female Nu/Nu nude mice were randomly separated into groups when the tumor volume reached 100 mm^3^_,_ and used to evaluate different treatments after intratumoral injection. To validate the generation of •OH in vivo, we first used the near‐infrared (NIR) fluorescence dye Cy7, which is degraded by •OH and therefore acts as a sensor. Tumor‐bearing mice received an intratumoral injection of CaO_2_@DOX@ZIF‐67 + Cy7 or Cy7 alone. In the group treated with Cy7 only, no obvious change in fluorescence was observed. In contrast, the fluorescence of the group treated with CaO_2_@DOX@ZIF‐67 + Cy7 decreased quickly (**Figure** 4a). It can be seen from Figure 4b that about 50% of the Cy7 was degraded within 6 h after intratumoral injection of CaO_2_@DOX@ZIF‐67 + Cy7. The results confirm the generation of •OH in the tumors.

**Figure 4:**
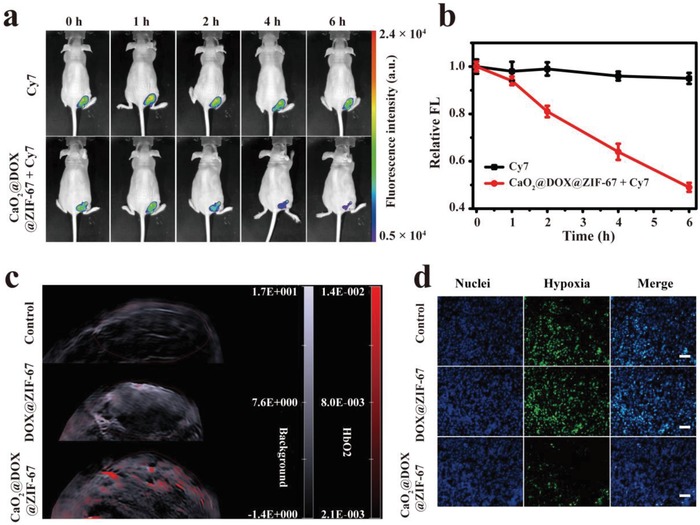
Confirm the production of •OH by detection of Cy7 degradation in vivo, a) fluorescence images and b) corresponding quantitative analysis of MCF‐7 tumor‐bearing mice with intratumoral injection of CaO_2_@DOX@ZIF‐67 + Cy7 or Cy7 alone, *n* = 3, mean ± SD; c) effect of saline (control), DOX@ZIF‐67, and CaO_2_@DOX@ZIF‐67 on tumor oxygenation: 2D photoacoustic images of MCF‐7 solid tumors in vivo at 12 h postinjection; d) HIF‐1α staining tumor tissues harvested from tumor‐bearing mice treated with saline, DOX@ZIF‐67, and CaO_2_@DOX@ZIF‐67. Green, HIF‐1α. Blue, DAPI. Scale bars are 100 µm.

Next, the oxygen‐generating capacity of CaO_2_@DOX@ZIF‐67 in tumors was evaluated by measuring oxygenated hemoglobin (HbO_2_) at 850 nm with a photoacoustic (PA) imaging system. Tumor‐bearing mice received an intratumoral injection of saline (control), DOX@ZIF‐67, and CaO_2_@DOX@ZIF‐67, respectively. As shown in Figure 4c, the PA signal intensity of HbO_2_ in the group treated with CaO_2_@DOX@ZIF‐67 is obviously higher. Compared with the saline (control) and DOX@ZIF‐67‐treated groups, the amount of intratumoral oxygen in the CaO_2_@DOX@ZIF‐67‐treated tumors increased approximately eightfolds and fourfolds, respectively (Figure S9, Supporting Information). These results were further verified by immunofluorescence staining assay using an antibody against hypoxia inducible factor‐1α (HIF‐1α) (Figure 4d). The group treated with PBS and DOX@ZIF‐67 displayed significant green fluorescence, which suggests that HIF‐1α is over expressed. However, owing to the elevated oxygen level in the tumor, the group treated with CaO_2_@DOX@ZIF‐67 only exhibited slight green fluorescence, which indicates that CaO_2_@DOX@ZIF‐67 can serve as an oxygen generator to overcome the hypoxia in tumor tissues.

We then used the xenograft model to evaluate the antitumor performance of different treatments after intratumor injection. Tumor‐bearing mice were administered with six treatments: saline (control), free DOX, DOX@ZIF‐67, CaO_2_@DOX, CaO_2_@ZIF‐67, and CaO_2_@DOX@ZIF‐67. In the groups treated with free DOX and DOX@ZIF‐67, there was modest inhibition of tumor growth compared to the control group (**Figure** 5a). These treatments do not generate O_2_ or H_2_O_2_; therefore the tumor cells will be insensitive to DOX, and there will be insufficient H_2_O_2_ for effective CDT. Tumor growth was obviously inhibited in the CaO_2_@DOX and CaO_2_@ZIF‐67 groups, and this inhibition possibly resulted from the generation of O_2_ and H_2_O_2_ from CaO_2_. Even so, tumor proliferation was not inhibited completely in these groups. The group treated with CaO_2_@DOX@ZIF‐67 exhibited the strongest tumor suppression effect, presumably due to the combined effect of chemotherapy and chemodynamic therapy. Images of representative tumors on day 21 after administration confirmed this result (Figure 5c). Hematoxylin–eosin (H&E) staining showed significant tumor cell necrosis and tumor tissue damage in the CaO_2_@DOX@ZIF‐67‐treated group compared with others (Figure 5d). Correspondingly, the terminal deoxynucleotidyl transferase (TdT) dUTP nick‐end labeling (TUNEL) staining assay also revealed a higher level of apoptosis (Figure 5d). Furthermore, during the treatment, no obvious body weight changes (Figure 5b) or major organ damage was observed in any of the groups (Figure S10, Supporting Information). This suggests that CaO_2_@DOX@ZIF‐67 has potential for clinical application.

**Figure 5:**
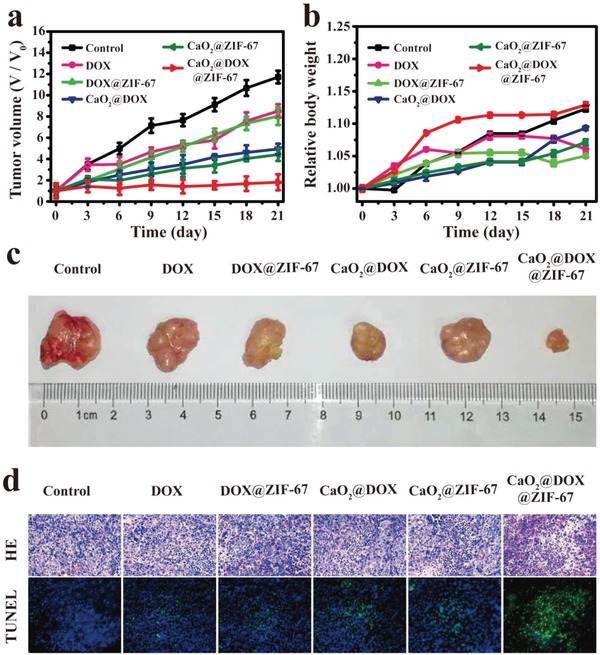
In vivo antitumor assay via intratumoral injection of various formulations. The changes of a) tumor volume and b) body weight of different groups of tumor‐bearing mice during treatment (*n* = 5, mean ± SD); c) representative photos of dissected tumors from the different groups on day 21 after administration; d) images of H&E and TUNEL stained sections of tumors from the different groups on day 21 after administration.

In summary, a pH‐triggered nanocatalytic medicine, CaO_2_@DOX@ZIF‐67, was successfully constructed via a bottom‐up approach for combined chemo/chemodynamic therapy of tumors. Under weakly acidic conditions, CaO_2_@DOX@ZIF‐67 can be broken down to rapidly release the Fenton‐like catalyst Co^2+^ and DOX. Subsequently, the unprotected CaO_2_ reacts with H_2_O to simultaneously generate O_2_ and H_2_O_2_, which will improve the efficacy of DOX and CDT, respectively. Both the in vitro and in vivo results demonstrated that CaO_2_@DOX@ZIF‐67 has an excellent antitumor performance and low systemic toxicity. Therefore, this nanocatalytic medicine could be a promising candidate for combined pH‐responsive chemo/chemodynamic therapy.

## Conflict of Interest

The authors declare no conflict of interest.

## Supporting information

Supporting Information

Supplemental Video 1

## References

[1] W. Fan , P. Huang , X. Chen , Chem. Soc. Rev. 2016, 45, 6488. 27722560 10.1039/c6cs00616g

[2] X. Qian , Y. Zheng , Y. Chen , Adv. Mater. 2016, 28, 8097. 27384408 10.1002/adma.201602012

[3] H. Ranji‐Burachaloo , P. A. Gurr , D. E. Dunstan , G. G. Qiao , ACS Nano 2018, 12, 11819. 30457834 10.1021/acsnano.8b07635

[4] a) B. Yang , Y. Chen , J. Shi , Chem. Rev. 2019, 119, 4881; 30973011 10.1021/acs.chemrev.8b00626

[5] Z. Tang , Y. Liu , M. He , W. Bu , Angew. Chem., Int. Ed. 2019, 58, 946. 10.1002/anie.201805664 30048028

[6] L. S. Lin , J. Song , L. Song , K. Ke , Y. Liu , Z. Zhou , Z. Shen , J. Li , Z. Yang , W. Tang , G. Niu , H. H. Yang , X. Chen , Angew. Chem., Int. Ed. 2018, 57, 4902. 10.1002/anie.201712027 29488312

[7] C. Zhang , W. Bu , D. Ni , S. Zhang , Q. Li , Z. Yao , J. Zhang , H. Yao , Z. Wang , J. Shi , Angew. Chem., Int. Ed. 2016, 55, 2101. 10.1002/anie.201510031 26836344

[8] M. Huo , L. Wang , Y. Chen , J. Shi , Nat. Commun. 2017, 8, 357. 28842577 10.1038/s41467-017-00424-8 PMC5572465

[9] L. H. Fu , C. Qi , J. Lin , P. Huang , Chem. Soc. Rev. 2018, 47, 6454. 30024579 10.1039/c7cs00891k

[10] S. Wang , Z. Wang , G. Yu , Z. Zhou , O. Jacobson , Y. Liu , Y. Ma , F. Zhang , Z. Y. Chen , X. Chen , Adv. Sci. 2019, 6, 1801986. 10.1002/advs.201801986 PMC6402284 30886808

[11] S. Gao , H. Lin , H. Zhang , H. Yao , Y. Chen , J. Shi , Adv. Sci. 2019, 6, 1801733. 10.1002/advs.201801733 PMC6364502 31168441

[12] W. Ke , J. Li , F. Mohammed , Y. Wang , K. Tou , X. Liu , P. Wen , H. Kinoh , Y. Anraku , H. Chen , K. Kataoka , Z. Ge , ACS Nano 2019, 13, 2357. 30699292 10.1021/acsnano.8b09082

[13] L. Feng , R. Xie , C. Wang , S. Gai , F. He , D. Yang , P. Yang , J. Lin , ACS Nano 2018, 12, 11000. 30339353 10.1021/acsnano.8b05042

[14] Y. Hu , T. Lv , Y. Ma , J. Xu , Y. Zhang , Y. Hou , Z. Huang , Y. Ding , Nano Lett. 2019, 19, 2731. 30919635 10.1021/acs.nanolett.9b01093

[15] Y. Liu , W. Zhen , Y. Wang , J. Liu , L. Jin , T. Zhang , S. Zhang , Y. Zhao , S. Song , C. Li , J. Zhu , Y. Yang , H. Zhang , Angew. Chem., Int. Ed. 2019, 58, 2407. 10.1002/anie.201813702 30600877

[16] D. Cioloboc , C. Kennedy , E. N. Boice , E. R. Clark , D. M. Kurtz, Jr. , Biomacromolecules 2018, 19, 178. 29192767 10.1021/acs.biomac.7b01433

[17] C. C. Huang , W. T. Chia , M. F. Chung , K. J. Lin , C. W. Hsiao , C. Jin , W. H. Lim , C. C. Chen , H. W. Sung , J. Am. Chem. Soc. 2016, 138, 5222. 27075956 10.1021/jacs.6b01784

[18] M. Ushio‐Fukai , Y. Nakamura , Cancer Lett. 2008, 266, 37. 18406051 10.1016/j.canlet.2008.02.044 PMC2673114

[19] Y. Dai , Z. Yang , S. Cheng , Z. Wang , R. Zhang , G. Zhu , Z. Wang , B. C. Yung , R. Tian , O. Jacobson , C. Xu , Q. Ni , J. Song , X. Sun , G. Niu , X. Chen , Adv. Mater. 2018, 30, 1704877. 10.1002/adma.201704877 29315862

[20] E. L. Samuel , D. C. Marcano , V. Berka , B. R. Bitner , G. Wu , A. Potter , R. H. Fabian , R. G. Pautler , T. A. Kent , A. L. Tsai , J. M. Tour , Proc. Natl. Acad. Sci. USA 2015, 112, 2343. 25675492 10.1073/pnas.1417047112 PMC4345556

[21] Y. Sheng , H. Nesbitt , B. Callan , M. A. Taylor , M. Love , A. P. McHale , J. F. Callan , J. Controlled Release 2017, 264, 333. 10.1016/j.jconrel.2017.09.004 28890213

[22] M. Z. Zou , W. L. Liu , C. X. Li , D. W. Zheng , J. Y. Zeng , F. Gao , J. J. Ye , X. Z. Zhang , Small 2018, 14, 1801120. 10.1002/smll.201801120 29882235

[23] B. Wu , L. Su , X. Dai , X. Chai , Chem. Eng. J. 2018, 335, 161.

[24] X. Long , Z. Yang , H. Wang , M. Chen , K. Peng , Q. Zeng , A. Xu , Ind. Eng. Chem. Res. 2012, 51, 11998.

[25] Y. Qian , J. Zhang , Y. Zhang , J. Chen , X. Zhou , Sep. Purif. Technol. 2016, 166, 222.

[26] C. Wang , S. Chen , Y. Wang , X. Liu , F. Hu , J. Sun , H. Yuan , Adv. Mater. 2018, 30, 1706407. 10.1002/adma.201706407 29484719

[27] E. Olyaie , H. Banejad , A. Afkhami , A. Rahmani , J. Khodaveisi , Sep. Purif. Technol. 2012, 95, 10.

[28] W. Zhang , X. Jiang , X. Wang , Y. V. Kaneti , Y. Chen , J. Liu , J. S. Jiang , Y. Yamauchi , M. Hu , Angew. Chem., Int. Ed. 2017, 56, 8435. 10.1002/anie.201701252 28382724

[29] M. Ni , B. D. Ratner , Surf. Interface Anal. 2008, 40, 1356. 25031482 10.1002/sia.2904 PMC4096336

[30] Z. Wang , Y. Zhang , Z. Tan , Q. Li , Chem. Eng. J. 2018, 350, 767.

[31] J. Qin , S. Wang , X. Wang , Appl. Catal., B 2017, 209, 476.

[32] H. Ranji‐Burachaloo , F. Karimi , K. Xie , Q. Fu , P. A. Gurr , D. E. Dunstan , G. G. Qiao , ACS Appl. Mater. Interfaces 2017, 9, 33599. 28885005 10.1021/acsami.7b07981

