# Correction to “Self‐Supply of O_2_ and H_2_O_2_ by a Nanocatalytic Medicine to Enhance Combined Chemo/Chemodynamic Therapy”

**DOI:** 10.1002/advs.76713

**Published:** 2026-07-20

S. Gao, Y. Jin, K. Ge, Z. Li, H. Liu, X. Dai, Y. Zhang, S. Chen, X. Liang, J. Zhang, “Self‐Supply of O_2_ and H_2_O_2_ by a Nanocatalytic Medicine to Enhance Combined Chemo/Chemodynamic Therapy.,” Advanced Science 6 no. 24, (2019): 1902137. https://doi.org/10.1002/advs.201902137

It has come to our attention that incorrect representative images were included in **Figure 5d** of the original version. Specifically, the TUNEL staining images for the CaO_2_@DOX and CaO_2_@ZIF‐67 groups in **Figure 5d** were inadvertently inserted incorrectly during figure assembly. The corrected Figure 5d, with group‐appropriate representative images and scale bars, is provided below. These corrections do not affect any of the experimental results, data interpretation, or conclusions reported in the paper.

In addition, the ethics approval number for the animal experiments was inadvertently omitted from the original article. The approval number is IACUC‐2018025.

We apologize for these errors.


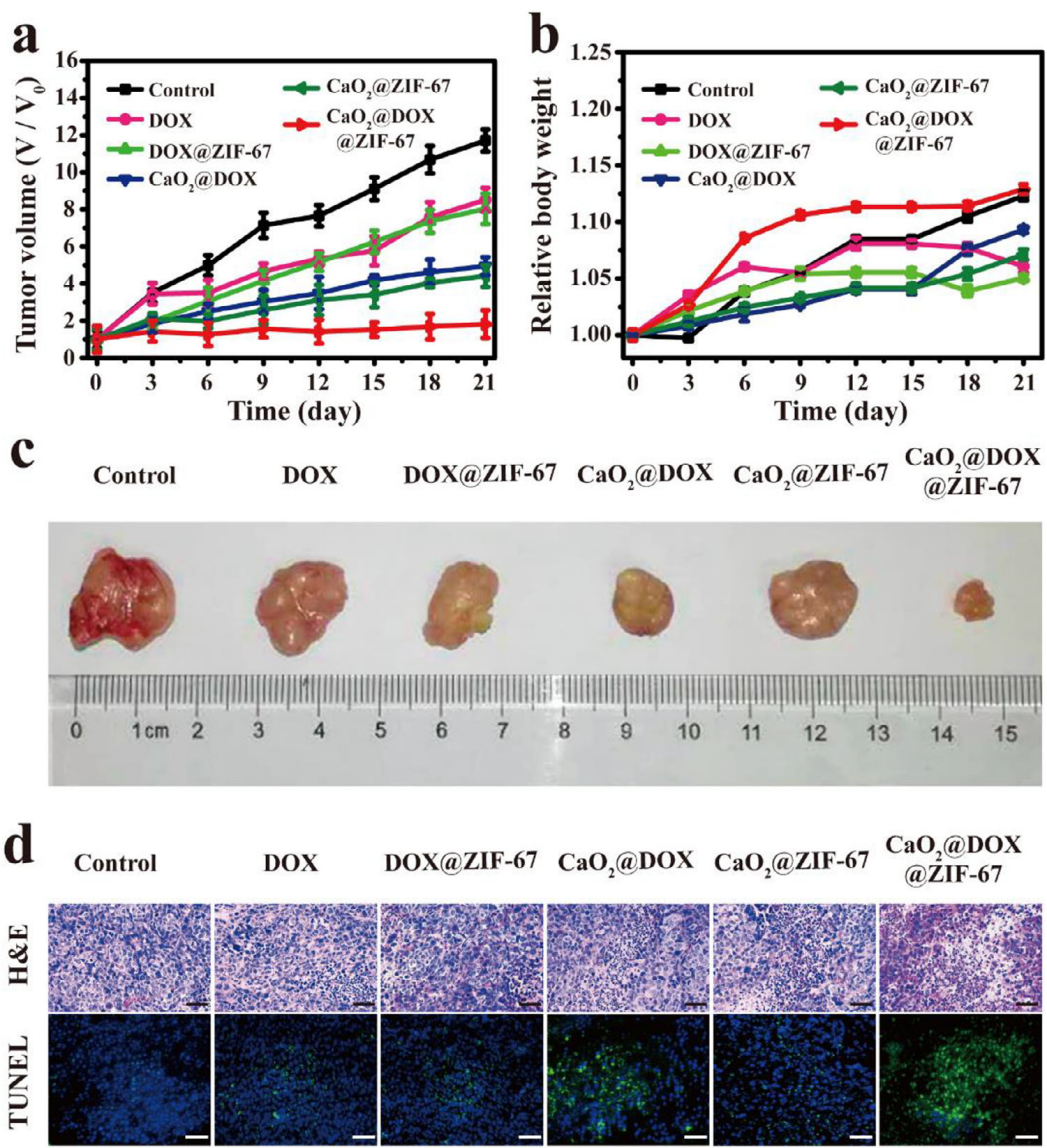


**Figure 5**. In vivo antitumor assay via intratumoral injection of various formulations. The changes of tumor volume (a) and body weight (b) of different groups of tumor‐bearing mice during treatment; (n = 5, mean ±SD); (c) Representative photos of dissected tumors from the different groups on day 21 after administration; (d) Images of H&E and TUNEL‐stained sections of tumors from the different groups on day 21 after administration. Scale bars are 50 µm.

