# Supplementary material for: Self‐Supply of O2 and H2O2 by a Nanocatalytic Medicine to Enhance Combined Chemo/Chemodynamic Therapy
Source: Adv Sci (Weinh). 2019 Oct 24;6(24):1902137. doi: 10.1002/advs.201902137 (PMC6918120; doi:10.1002/advs.201902137)
Supplement: Supplementary file 1 — Supporting Information [file ADVS-6-1902137-s001.pdf]

Copyright WILEY-VCH Verlag GmbH & Co. KGaA, 69469 Weinheim, Germany, 2019.

ADVANCED SCIENCE
Open Access

# Supporting Information

for *Adv. Sci.,* DOI: 10.1002/advs.201902137

## Self-Supply of $O_2$ and $H_2O_2$ by a Nanocatalytic Medicine to Enhance Combined Chemo/Chemodynamic Therapy

*Shutao Gao, Yan Jin, Kun Ge, Zhenhua Li,* Huifang Liu, Xinyue Dai, Yinghua Zhang, Shizhu Chen, Xingjie Liang,* and Jinchao Zhang**

# Supporting Information

## Self-supply of $O_2$ and $H_2O_2$ by a Nanocatalytic Medicine to Enhance Combined Chemo/Chemodynamic Therapy

*Shutao Gao[1, 2,#],Yan Jin[1,#], Kun Ge[1], Zhenhua Li[1,]*, Huifang Liu[1], Xinyue Dai[1], Yinghua Zhang[1], Shizhu Chen[3], Xing-Jie Liang[3,]* and Jinchao Zhang[1,]**

[1]College of Chemistry& Environmental Science, Analytical Chemistry Key Laboratory of Hebei Province, Chemical Biology Key Laboratory of Hebei Province, Key Laboratory of Medicinal Chemistry and Molecular Diagnosis of the Ministry of Education, Hebei University, Baoding 071002,P. R. China.

[2]College of Science, Hebei Agricultural University, Baoding 071001, P. R. China.

[3]CAS Key Laboratory for Biological Effects of Nanomaterials and Nanosafety, National Center for Nanoscience and Technology, Beijing 100190, P. R. China.

Correspondence and requests for materials should be addressed to Z.L. (email: zhenhuali@hbu.edu.cn), X.L. (email: liangxj@nanoctr.cn) or to J.Z. (email: jczhang6970@163.com).

# These authors contributed equally to this work.

## Materials

Calcium chloride ($CaCl_2$), hydrogen peroxide ($H_2O_2$, 30 wt%), ammonia solution ($NH_3 \cdot H_2O$, 25 wt%), polyethylene glycol 200 (PEG 200), cobalt nitrate hexahydrate ($Co(NO_3)_2 \cdot 6H_2O$), 2-methylimidazole, methanol and triethylamine were all purchased from Huaxin Co., Ltd. (Baoding, China). Doxorubicin hydrochloride (DOX), 2′,7′-dichlorofluorescin diacetate (DCFH-DA) and 3,3′,5,5′-tetramethylbenzidine (TMB, 99%) were purchased from Sigma-Aldrich (USA). Dulbecco's modified Eagle's medium (DMEM) and fetal bovine serum (FBS) were purchased from

Invitrogen. The human breast adenocarcinoma cell line MCF-7 was purchased from the Cell Bank of the Chinese Academy of Sciences (Shanghai).

**Physicochemical Characterization**

The morphology of the as-prepared nanocatalytic medicine was observed by transmission electron microscopy (TEM, TecnaiG$^2$F20S-TWIN). The size distribution was measured by a Zetasizer Nano ZS (ZEN3600, Malvern Instruments). The Brunauer–Emmett–Teller (BET) surface area was measured using an APP V-Sorb 2800P Surface Area and Porosity Analyzer (Jinaipu, China). UV-vis absorption spectra and absorbance were recorded on a UV–vis spectrometer (UV-2600, Shimadzu). The X-ray diffraction (XRD) patterns of the samples were recorded with an X-ray powder diffractometer using Cu Ka radiation (D8ADVANCE, Bruker, Switzerland) in the range $2\theta = 2^o$-$60^o$. The ESR spectrum of the as-prepared sample was measured using a Bruker EMX1598 spectrometer. 5,5-Dimethyl-1-pyrroline-N-oxide (DMPO) was selected as the nitrogen trapping agent to evaluate the generation of hydroxyl radicals (•OH). The composition of the evolved gas from $CaO_2$@DOX@ZIF-67 was confirmed by gas chromatography (thermal cond uctivity detector).

**Synthesis of $CaO_2$ Nanoparticles**

$CaO_2$ nanoparticles were synthesized according to the previous literature with a slight modification.[1] Typically, 1 g $CaCl_2$ was dissolved in 10 mL distilled water, then 5 mL of 1 mol/L ammonia solution and 80 mL of PEG 200 were added in sequence under vigorous stirring to give a clear solution. Subsequently, 5 mL of 30% $H_2O_2$ was

added dropwise to this solution within 20 min under constant stirring. After that, the mixture was centrifuged at 9000 rpm for 15 min and washed three times with methanol. The white $CaO_2$ product was dried under vacuum at 50 °C for 4 h.

**Synthesis of $CaO_2$@DOX**

The as-prepared $CaO_2$ (50 mg) was dispersed into 40 mL methanol under ultrasonic wave treatment. Then, 10 mL DOX/methanol solution (1 mg $mL^{-1}$) was added and the mixture was stirred for 10 h. Finally, the sample was centrifuged at 9000 rpm for 10 min and washed three times with methanol. The purple $CaO_2$@DOX product was obtained after drying under vacuum at 50 °C for 4 h.

**Synthesis of $CaO_2$@DOX@ZIF-67**

$CaO_2$@DOX@ZIF-67 was synthesized via *in situ* assembly. Firstly, 50 mg $CaO_2$@DOX was dispersed into 20 mL methanol under ultrasonic wave treatment, and then 12 mg $Co(NO_3)_2$•$6H_2O$ was dissolved into the above solution to give a mixture (denoted as A). Secondly, 13 mg 2-methylimidazole and 6 μL triethylamine were dissolved into 20 mL methanol under ultrasonic wave treatment to give a clear solution (denoted as B). Lastly, solution B was poured into solution A and stirred for a certain time until the color turned to olivine. The products were isolated by centrifugation and washed three times with methanol. The olivine $CaO_2$@DOX@ZIF-67 product was obtained after drying under vacuum at 50 °C for 4 h.

**Generation of •OH by $CaO_2$@DOX@ZIF-67**

The generation of •OH was determined by ESR spectroscopy with 5, 5-dimethyl-1-pyrroline N-oxide (DMPO) as a spin trap for the hydroxyl radical. In detail, 0.5 mg $CaO_2$@DOX@ZIF-67 was added into 60 μL of DMPO buffer solution (100 mM) with different pH values (pH = 5.0, 6.5 and 7.4). ESR spectroscopy was then immediately performed on the mixture using a JES FA200 spectrometer.

The chromogenic reaction of TMB was further used to verify that the production of •OH is dependent on the concentration of $CaO_2$@DOX@ZIF-67. Typically, 50 μL of TMB (0.1 μM) was added into 3 mL acetate buffer solution (0.1 M, pH 5.0), then various amounts of $CaO_2$@DOX@ZIF-67 were dispersed into this solution and stirred for 5min. UV-vis absorption spectra were then recorded by a UV-vis spectrometer (UV-2600,Shimadzu). The total amount of $H_2O_2$ generated from $CaO_2$@DOX@ZIF-67 was quantitatively determined according to the UV-vis absorbance standard curve of ox-TMB at 370 nm.

**Generation of $O_2$ by $CaO_2$@DOX@ZIF-67**

20 mg $CaO_2$@DOX@ZIF-67 was dispersed into 20 mL deoxygenated acetate buffer solution (0.1 M, pH 5.0), and then the oxygen concentration was monitored every minute for 10 min with a portable dissolved oxygen meter.[2] As a control, the oxygen concentration of 20 mL deoxygenated water was monitored under the same conditions.

***In Vitro* Drug Release**

The *in vitro* release of DOX from $CaO_2$@DOX@ZIF-67 was determined with UV−vis spectrophotometry.[3] Typically, 10 mg of $CaO_2$@DOX@ZIF-67 was dispersed in

20.0 ml of buffer solution at pH = 7.4, 6.5, and 5.0. The release experiment was carried out at 37°C under stirring. At regular intervals, 2 mL of the suspension was withdrawn and centrifuged. The absorbance of the supernatant at 480 nm was measured by a UV-vis spectrometer (UV-2600, Shimadzu), and then the mixture was returned to the original suspension. The total amount of loaded DOX was quantitatively determined according to the standard curve of UV-vis absorbance at 480 nm. The DOX release percentages were calculated via the formula: release percentage (%) = ($m_1$/$m_2$) x 100, where $m_1$ is the amount of released DOX, and $m_2$ is the total amount of loaded DOX.

**$H_2O_2$ release assay**

The production of $H_2O_2$ was verified through the formation of yellow titanium peroxide complex ($TiO_2^{2+}$) using $H_2O_2$ assay kit obtained from Solarbio Life Sciences. All the experiments followed the protocol of Solarbio Life Sciences using $CaO_2$@DOX@ZIF-67 instead of cell extract.

**Cell Culture**

MCF-7 cells were cultured in DMEM medium with 10% FBS and 1% antibiotics (penicillin-streptomycin, 10 000 UmL-1) at 37°C in a humidified atmosphere containing 5% CO2.[4]

**Culture of MCF-7 Multicellular Tumor Spheroids (MCTSs)**

For the formation of MCF-7 multicellular tumor spheroids (MCTSs), MCF-7 cells were seeded in ultralow-attachment 12-well plates at a density of 2000 cells per well and cultured in DMEM/F12 medium supplemented with10 ng mL−1 bFGF, 20 ng

mL−1 EGF, 1% N2, and 2% B27. After culturing for 10-12 days, further experiments were conducted using the as-prepared MCTSs.[5]

***In Vitro* Cell Experiments**

The MTT assay was used to evaluated the *in vitro* cytotoxicity of $CaO_2$@DOX@ZIF-67.[6] MCF-7 cells were cultured in a 96-well plate at a density $4\times10^3$ cells per well for 24 h at 37 °C. After that, the medium was replaced by fresh DMEM medium containing different concentrations of $CaO_2$@DOX@ZIF-67 (10 μg $mL^{-1}$, 20 μg $mL^{-1}$, 30 μg $mL^{-1}$, 40 μg $mL^{-1}$, 50 μg $mL^{-1}$). The pH value was then adjusted to 7.4 or 6.5 by adding diluted HCl. After incubation for 12 h, 20 μL MTT was added into each well and further incubated for 4 h. Subsequently, the MTT (5 mg $mL^{-1}$) was replaced by 150 μL of dimethylsulfoxide (DMSO). The absorbance of each well was measured using a microplate reader at 570 nm. The relative cell viability was determined by comparing with the control.

In addition, the cytotoxicity of various samples (free DOX, $CaO_2$@DOX, DOX@ZIF-67, $CaO_2$@ZIF-67 and $CaO_2$@DOX@ZIF-67) in normoxic (21% $O_2$) or hypoxic (1% $O_2$) conditions was also evaluated by MTT assay. Free DOX, $CaO_2$@DOX, DOX@ZIF-67 and $CaO_2$@DOX@ZIF-67 were added with equal DOX concentrations (2.5 μg $mL^{-1}$). The concentration of $CaO_2$@ZIF-67 was the same as $CaO_2$@DOX@ZIF-67 (50 μg $mL^{-1}$).

**Intracellular ·OH Production[7]**

In order to verify the generation of intracellular ROS by $CaO_2$@DOX@ZIF-67, MCF-7 cells were seeded in confocal dishes. After culture for a certain time, 100 μL

of saline, DOX@ZIF-67 (50 μg $mL^{-1}$) or $CaO_2$@DOX@ZIF-67 (50 μg $mL^{-1}$) was added into the medium. After incubation for 12 h, the cells were rinsed carefully with PBS. Then 1.8 mL of PBS and 200 μL of DCFH-DA (0.1M) was added and incubated for an additional 30 min. Finally, the fluorescence of DCF ($\lambda_{ex}$ = 488 nm, $\lambda_{em}$= 525 nm) was observed with a confocal laser scanning microscope to evaluate the level of intracellular ROS.

**Co-localization Experiment**[8]

$CaO_2$@DOX@ZIF-67 was labeled with FITC. Briefly, 1mL FITC/ethanol solution (1 mg $mL^{-1}$) was mixed with 10 mL $CaO_2$@DOX@ZIF-67/ethanol solution (1 mg $mL^{-1}$). The mixture was stired for 10 h in dark at room temperature. Then the mixture was centrifuged at 9000 rpm for 10 min and washed three times with ethanol to obtained FITC-labeled $CaO_2$@DOX@ZIF-67. Subsequently, the location experiment of $CaO_2$@DOX@ZIF-67 in MCF-7 cell was conducted. MCF-7 cells were incubated with FITC-labeled $CaO_2$@DOX@ZIF-67 (50 μg $mL^{-1}$) at 37 °C for 4 h. Subsequently, the cells were co-cultured with Lyso Tracker Red (100 nM) and Hoechst 33342 (100 nM) at 37 °C for 20 min, respectively. After culturing, MCF-7 cells were washed by PBS for three times. And then , the cells were subjected to the confocal fuorescence imaging measurements.

**Efficacy of Combined Chemo/chemodynamic Therapy in MCTSs**

The live/dead staining assay was used to evaluate the efficacy of $CaO_2$@DOX@ZIF-67 to kill cells in MCTSs.[9] The MCTSs were incubated with DOX@ZIF-67 (50 μg $mL^{-1}$) and $CaO_2$@DOX@ZIF-67 (50 μg $mL^{-1}$) for 12 h in

6-well plates. MCTSs incubated with saline served as control. Then the MCTSs were rinsed carefully with PBS for three times. After that, 2 mL calcein-AM (2 μM) and propidium iodide (PI, 4 μM) solution was added into the 6-well plates and further incubated for 15 min. The MCTSs were washed with PBS, then imaged with a fluorescence microscope (Life Technologies; EVOS FL, USA).

**Establishment of MCF-7 xenograft model mice for *in vivo* experiments**

All animal experiments procedures comply with the guidelines of the Institutional Animal Care and Use Committee (IACUC) and the care regulations approved by the Animal Welfare and Ethical Committee of Hebei University. The MCF-7 xenograft tumor model was established by subcutaneously injecting $5\times10^6$ MCF-7 cells into female Nu/Nu nude mice at 4~5 weeks of age (~20 g). Once the tumors grow to ~100 $mm^3$, the mice were randomly divided into groups for the following experiments.

***In Vivo* Photoacoustic (PA) Imaging Experiments**

MCF-7 tumor-bearing mice were randomly divided into three groups: saline, DOX@ZIF-67 and $CaO_2$@DOX@ZIF-67 (n = 3 per group). The formulations (25 μL, 1 mg $mL^{-1}$) were injected into the tumors of the tumor-bearing mice. After 4 h, the oxygenated hemoglobin signal at 850 nm was obtained and analyzed with a Photoacoustic Imaging System (MSOT in Vision 128).[10]

**Intratumoral ·OH Production**

In order to verify that $CaO_2$@DOX@ZIF-67 induced the production of ROS in tumors, MCF-7 tumor-bearing mice were randomly dividedinto two groups(n = 3 per group). In group 1, tumors were injected with 10 μL of Cy 7 (0.5μg $mL^{-1}$). In group 2,

tumors were injected with 15 μL of $CaO_2$@DOX@ZIF-67 (1 mg $mL^{-1}$) and 10 μL of Cy 7 (0.5μg $mL^{-1}$). An IVIS Spectrum Imaging System (Perkin Elmer Inc) was used to capture fluoresence images of the mice at 0 h, 1 h, 2 h, 4 h and 6 h time-points.

**HIF-1α Staining**

MCF-7 tumor-bearing mice were randomly divided into three groups (n = 3 per group), and then the mice received an intratumoral injection of 25 μL of saline, DOX@ZIF-67 (with a DOX concentration 0.25 mg $Kg^{-1}$) and $CaO_2$@DOX@ZIF-67 (with a DOX concentration 0.25 mg $Kg^{-1}$). 24 h post-injection, all the mice were sacrificed and tumor tissues were collected and fixed in 4% formalin for staining with an anti-HIF-1α antibody.

***In Vivo* Antitumor Assay**

MCF-7 tumor-bearing mice were randomly divided into six groups (each group containing five mice): (1) saline, (2) DOX, (3) $CaO_2$@DOX, (4) DOX@ZIF-67, (5) $CaO_2$@ZIF-67 and (6) $CaO_2$@DOX@ZIF-67. Group 1 received 25 μL of saline viaintratumoral injection; group 5 received 25 μL of $CaO_2$@ZIF-67 via intratumoral injection at a dose of 5 mg $Kg^{-1}$; and groups 2-4 were respectively treated with DOX, $CaO_2$@DOX, DOX@ZIF-67, and $CaO_2$@DOX@ZIF-67 via intratumoral injection with equivalent amounts of DOX (0.25 mg $Kg^{-1}$ of mouse body weight). After these treatments, the tumor volume and body weight of each mouse were monitored every 3 days. The tumor volume was calculated using the formula: Volume = $Width^2 \times Length/2$. The relative tumor volume was obtained using the formula: Relative Volume = Measured Volume/ Initial Volume. All the mice were euthanized after 21 d

treatment. The major organs and tumors were harvested for further H&E staining and TUNEL assays.

## References

[1] Y. Qian, J. Zhang, Y. Zhang, J. Chen, X. Zhou, *Sep. Purif. Technol.* **2016**, *166*, 222.

[2] G. Song, C. Liang, X. Yi, Q. Zhao, L. Cheng, K. Yang, Z. Liu, *Adv. Mater.* **2016**, *28*, 2716.

[3] Y. Dai, Z. Yang, S. Cheng, Z. Wang, R. Zhang, G. Zhu, Z. Wang, B. C. Yung, R. Tian, O. Jacobson, C. Xu, Q. Ni, J. Song, X. Sun, G. Niu, X. Chen, *Adv. Mater.* **2018**, *30*, 1704877.

[4] S. Gao, P. Zheng, Z. Li, X. Feng, W. Yan, S. Chen, W. Guo, D. Liu, X. Yang, S. Wang, X. J. Liang, J. Zhang, *Biomaterials* **2018**, *178*, 83.

[5] H. Li, W. Yan, X. Suo, H. Peng, X. Yang, Z. Li, J. Zhang, D. Liu, *Biomaterials* **2019**, *200*, 1.

[6] L. Feng, R. Xie, C. Wang, S. Gai, F. He, D. Yang, P. Yang, J. Lin, *ACS nano* **2018**, *12*, 11000.

[7] P. Hu, T. Wu, W. Fan, L. Chen, Y. Liu, D. Ni, W. Bu, J. Shi, *Biomaterials* **2017**, *141*, 86.

[8] a) M. Li, J. Xia, R. Tian, J. Wang, J. Fan, J. Du, S. Long, X. Song, J. W. Foley, X. Peng, *J. Am. Chem. Soc.* **2018**, *140*, 14851; b) Q. Zheng, W. Cheng, X. Zhang, R. Shao, Z. Li, *Nanoscale Res. Lett.* **2019**, *14*, 305

[9] Y. Wang, Y. Xie, J. Li, Z. H. Peng, Y. Sheinin, J. Zhou, D. Oupicky, *ACS nano* **2017**, *11*, 2227.

[10] P. Prasad, C. R. Gordijo, A. Z. Abbasi, A. Maeda, A. Ip, A. M. Rauth, R. S. DaCosta, X. Y. Wu, *ACS nano* **2014**, *8*, 3202.

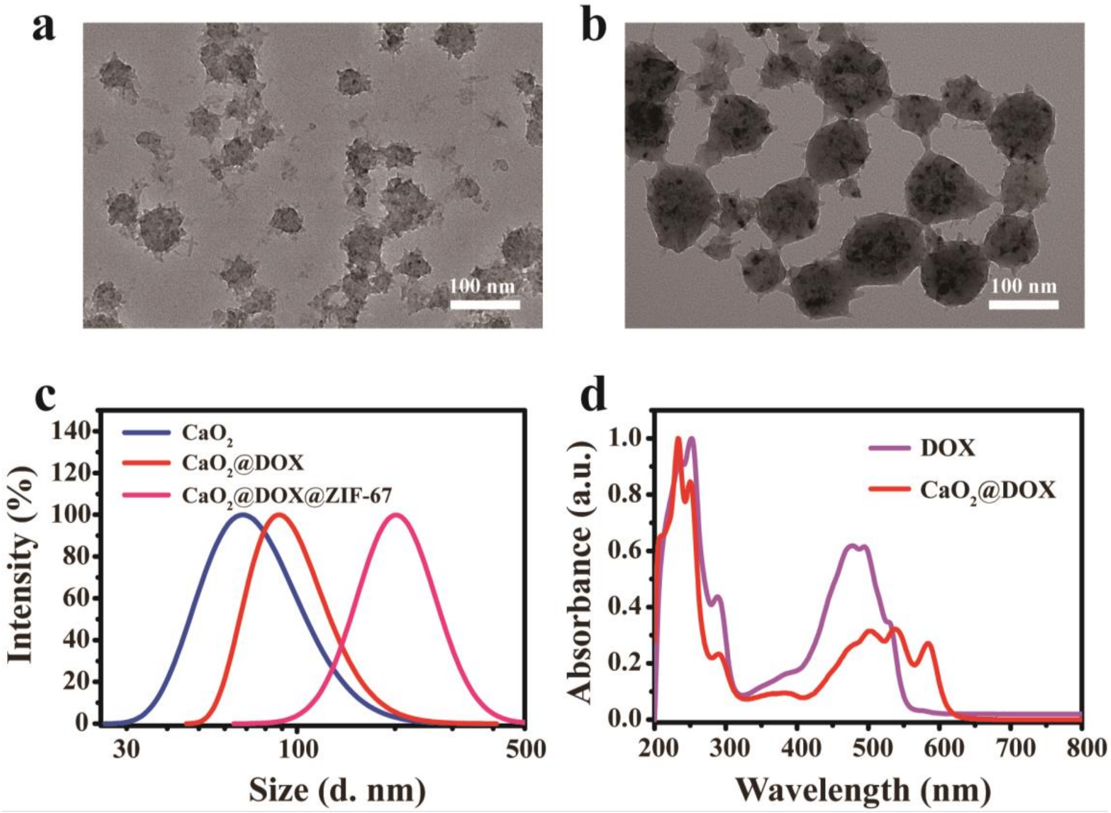


**Figure S1**. TEM images of (a) $CaO_2$ and (b) $CaO_2$@DOX; (c) Size distribution of $CaO_2$, $CaO_2$@DOX and $CaO_2$@DOX@ZIF-67 measured by DLS; (d) UV-vis absorption spectrum of DOX and $CaO_2$@DOX.

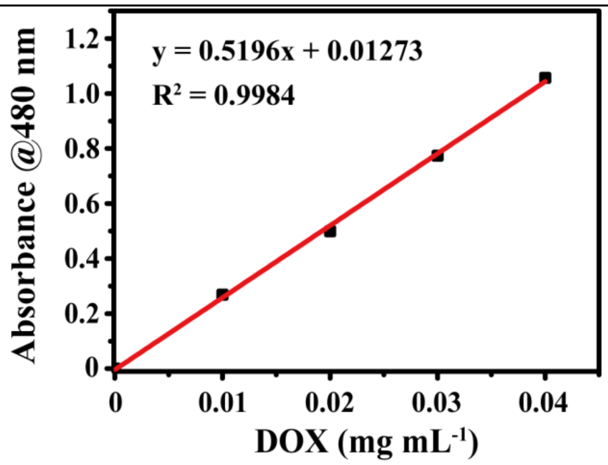


**Figure S2**. Standard curve to determine DOX release.

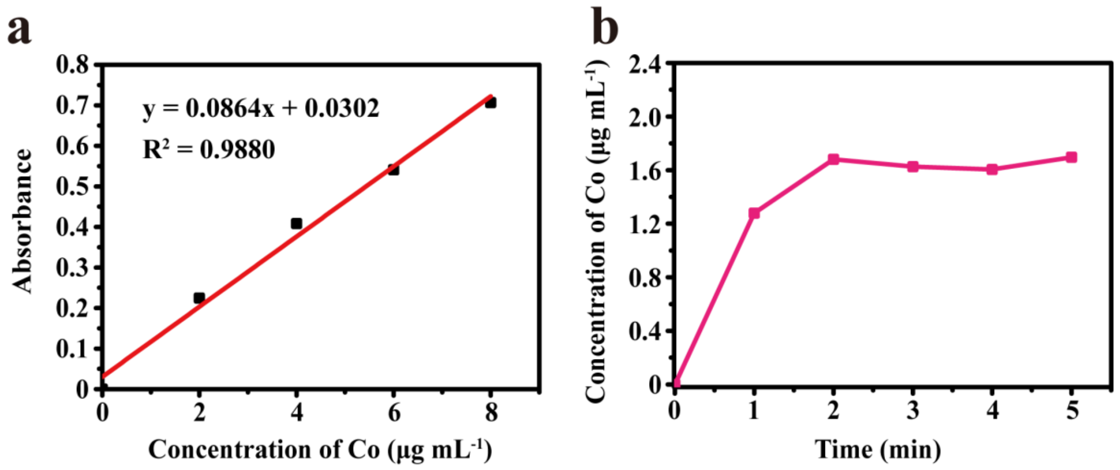


**Figure S3**. (a) Standard curve to determine $Co^{2+}$ release. (b) The release of $Co^{2+}$ resulted from the degradation of ZIF-67 in acetate buffer. 5 mg ZIF-67 was dispersed in 25 mL of acetate buffer (pH 5.0). At the given time interval, 1 mL suspension was withdrawn and filtered to remove the remained particles. The concentration of $Co^{2+}$ was determined with SHIMADZU AA-6300C atomic absorption spectrophotometer.

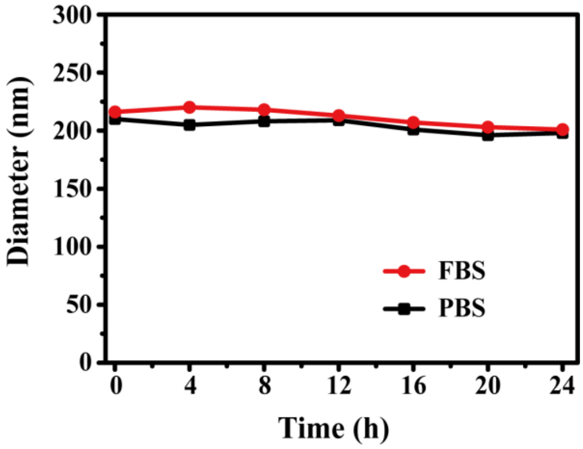


**Figure S4**. Stability of $CaO_2$@DOX and ZIF-67 nanoparticles in 1× PBS and 100% FBS within 24 h.

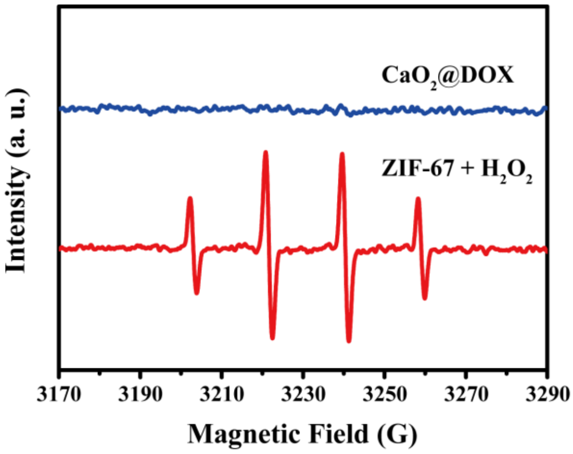


**Figure S5.** ESR spectra of $CaO_2$@DOX and ZIF-67 + $H_2O_2$ at pH 5.0 with 5,5-dimethyl-1-pyrroline N-oxide (DMPO) as the spin trap.

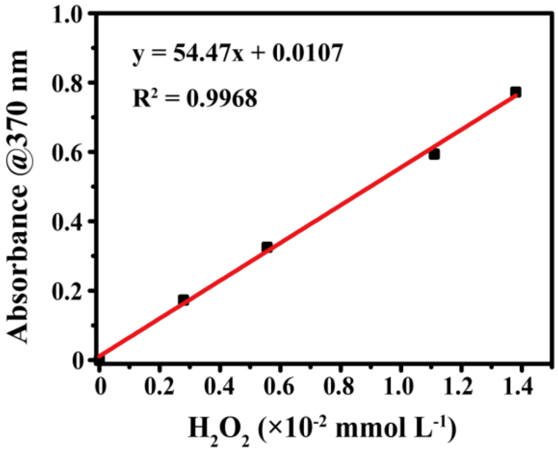


**Figure S6**. Standard curve to determine $H_2O_2$ release.

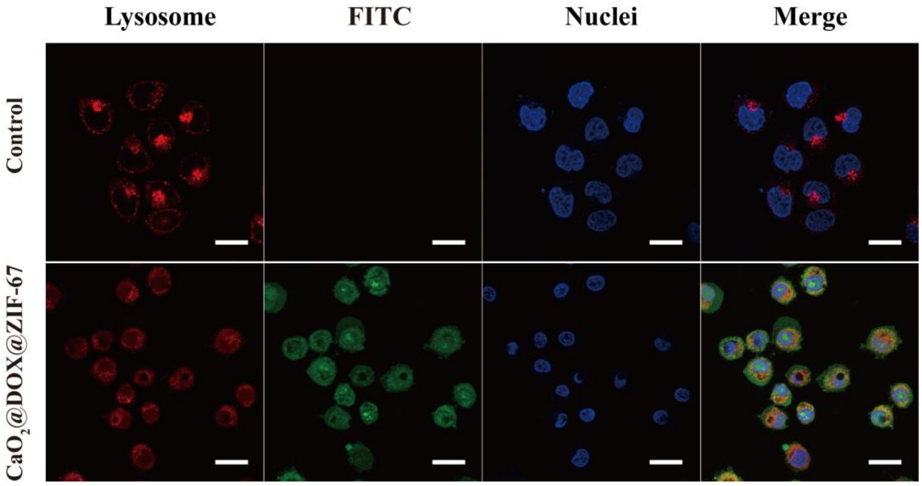


**Figure S7**. Confocal fluorescence microscope observation intracellular localization of $CaO_2$@DOX@ZIF-67. Red fluorescence: Lyso Tracker Red; Green fluorescence: FITC-labeled $CaO_2$@DOX@ZIF-67; Blue fluorescence: Hoechst 33342. The scale bar is 20 μm.

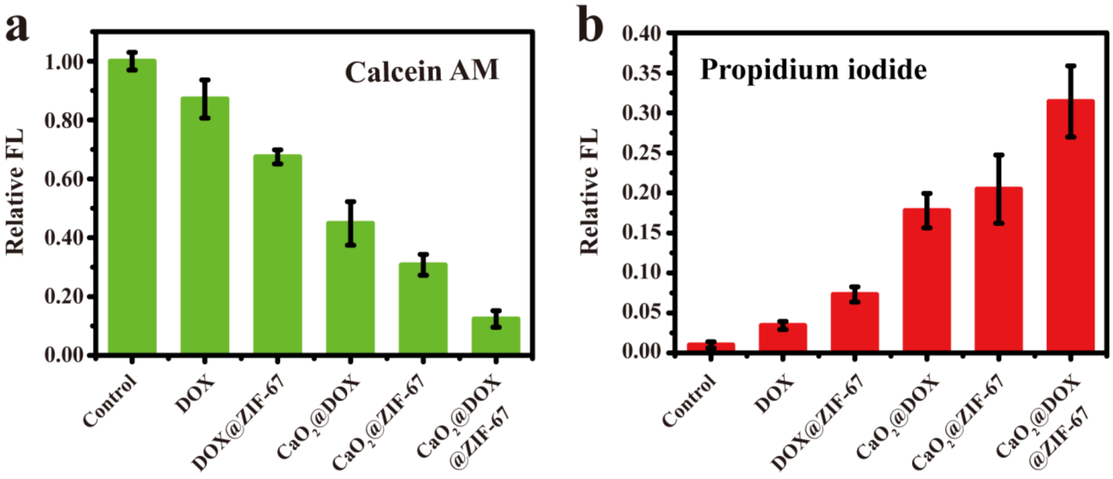


**Figure S8**. Statistical analysis of the fluorescence intensity of live/dead cell assays in MCF-7 MCTSs with various formulations treatments using ImageJ software.

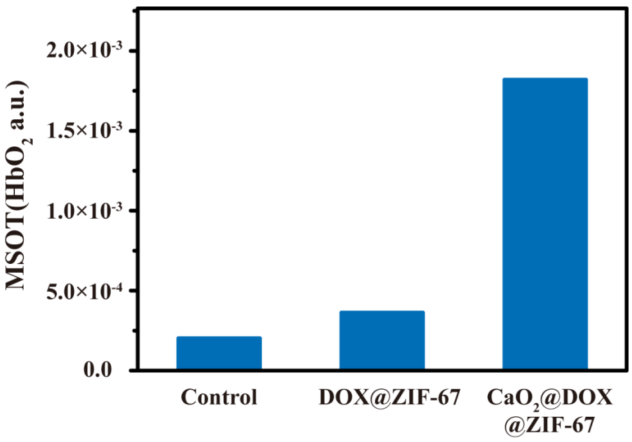


**Figure S9**. Comparison of the average $HbO_2$ signal intensity in tumors from mice receiving different treatments.

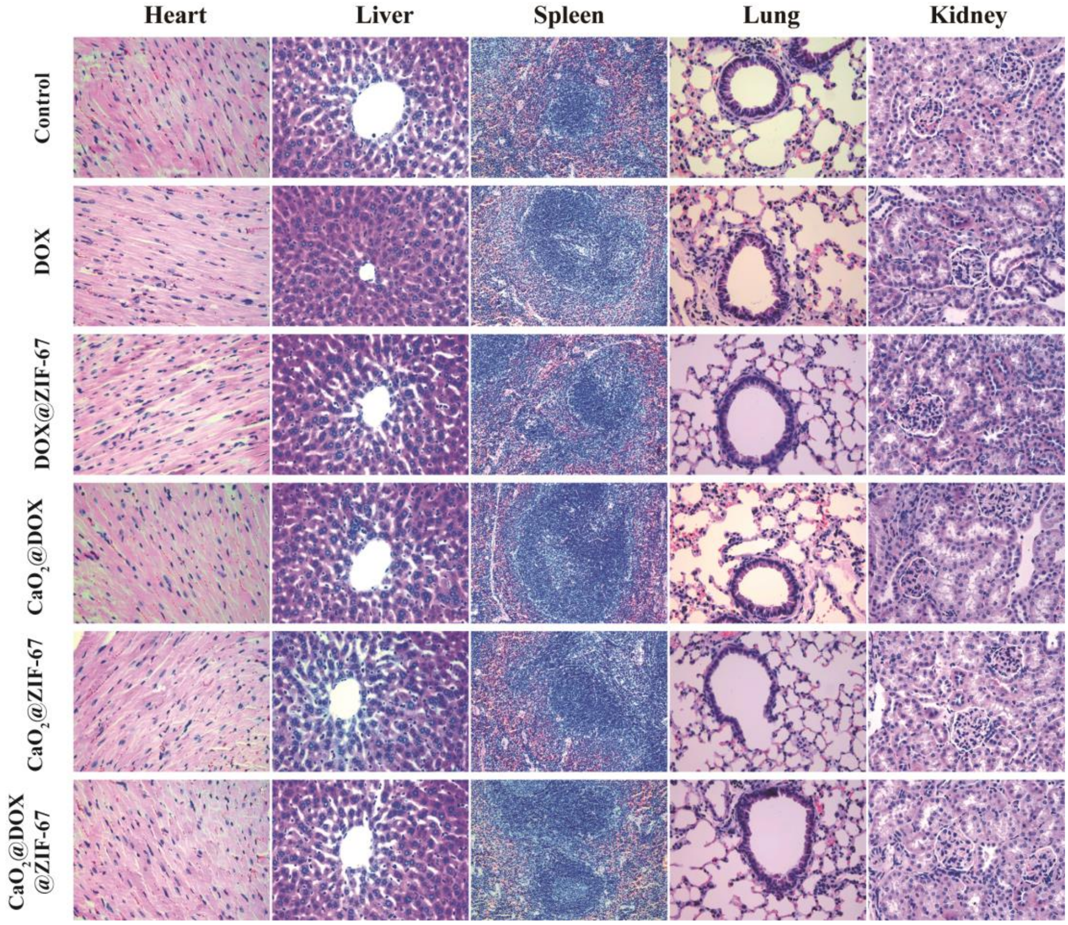


**Figure S10**. Images of H&E-stained sections of the heart, liver, spleen, lung and kidney from mice in each treatment group.
